# Overall survival in patients over 40 years old with surgically resected pancreatic carcinoma: a SEER-based nomogram analysis

**DOI:** 10.1186/s12885-019-5958-9

**Published:** 2019-07-23

**Authors:** Jian Li, Leshan Liu

**Affiliations:** Clinical Research Center, Ruijin Hospital, Shanghai Jiao Tong University School of Medicine, 197 Ruijin Er Road, Huangpu District, Shanghai, 200025 China

**Keywords:** Pancreatic carcinoma, Prognosis, Overall survival, Nomogram

## Abstract

**Background:**

The aim of this study was to identify the determinants of overall survival (OS) within patients over 40 years old with surgically resected pancreatic carcinoma (PC), and to develop a nomogram with the intention of OS predicting.

**Methods:**

A total of 6341 patients of 40 years of age or later with surgically resected PC between 2010 and 2015 were enrolled from the Surveillance, Epidemiology, and End Results (SEER) program and randomly assigned into training set (4242 cases) and validation set (2099 cases). A nomogram was constructed for predicting 1-, 2- and 3-years OS based on univairate and multivariate Cox regression. The C-index and calibration plot were adopted to assess the nomogram performance.

**Results:**

Our analysis showed that age, location of carcinoma in pancreas, tumor grade, TNM stage, size of carcinoma together with lymph node ratio (LNR) were considered to be independent overall survival predictors. A nomogram based on these six factors was developed with C-index being 0.680 (95%CI: 0.667–0.693). All calibration curves of OS fitted well. The OS curves stratified by nomogram-predicted probability score (≥20, 10–19 and < 10) demonstrated statistically significant difference not only within training set but also in validation set.

**Conclusions:**

The present nomogram for OS predicting can serve as the efficacious survival-predicting model and assist in accurate decision-making for patients over 40 years old with surgically resected PC.

## Background

Pancreas carcinoma (PC), an extraordinarily common cancer, ranks as the fourth leading cause of cancer death in the western countries [1]. The morbidity and mortality of PC have been on the rise currently, and its morbidity shows a youth oriented tendency. Most of PC patients are older than 40 years of age. Worldwide, PC accounts for more than 200 000 deaths annually. Moreover, it is anticipated to become the second dominating death cause in malign neoplasms by 2030 [2]. In spite of great progresses in surgery, neoadjuvant chemoradiotherapy and immunotherapy, PC prognosis still remains dismal with the overall survival (OS) of 5-year hovering at 8% [3]. The potentially curative therapy for PC patient is surgical resection. Nevertheless, merely 20% of PC patients are potentially curative resected candidates owing to difficulty in early diagnosis [4], and the prognosis of long-term is poor [5]. Among patients undergoing radical resection, recurrence will occur in most patients ultimately. Hence, clinicopathologic-based, personalized prognostic evaluation of PC patients can be in favor of undertaking superior therapeutic strategies.

Since PC is heterogeneous with respect to survival of individual patients, it is necessitated to develop a more personalized prognostic tool which may offer the precise survival prediction for these patients. Presently, the staging system of Tumor-Node-Metastasis (TNM) derived from the 8th edition of American Joint Commission on Cancer (AJCC), formulated for prognostic predicting after surgical resection, is one of the most widely adopted predictor of cancer prognosis [6, 7]. The TNM classification system only takes carcinoma size and extent, presence of lymph nodes metastasis and distant recurrence into account. Actually, other vital non-TNM indicators like gender, age, marital status, serum carbohydrate antigen 19–9 (CA19–9) and tumor differentiation have already been found to associate with PC patient survival [8–10]. In addition, the lymph node ratio (LNR) demonstrated an impact on prognosis [11], and could serve as a active predictor for survival [12, 13]. Therefore, a more precise predicting system is needed to establish to assist clinicians in making individual survival prediction.

Currently, nomograms have been developed and proposed as a novel, alternative tool for prognostic evaluation of many cancers [14–16], which can incorporate important demographic and clinicopathologic characteristics to estimate the individual survival rate for cancer patients. Since PC rarely occurs before the age of 40, a nomogram for PC patients 40 years of age or older undergoing surgical resection derived from population-based data, to our knowledge, has not ever been reported. We aim to formulate a prognostic nomogram with the data from Surveillance, Epidemiology and End Results (SEER) of the US National Cancer Institute (NCI) to better predict individualized prognosis in surgically resected PC patients who are age 40 or older.

## Methods

### Patient population

Data of this study were retrieved from the SEER program, which covered up to 97% of incidence of cancer and encompassed 28% of the US population [17], and accessed by SEER*Stat software v. 8.3.5. Inclusion criteria indicated below: 1) Patients were diagnosed with PC as the first and sole carcinoma diagnosis and diagnosing age were ≥ 40 years old. 2) Those with a confirmed pathological diagnosis from 2010 to 2015 and undergoing surgical resection.3) Site of pancreatic neoplasm (primary site-labeled) was limited to the site code of C25.0, C25.1 and C25.2 from the International Classification of Diseases for Oncology, 3rd Edition (ICD-O-3). 4) Active following-up with clear data and known outcome. Exclusion criteria were as follows: 1) Patients with second primary carcinoma. 2) Those diagnosed with AJCC TNM stage III or IV who were thought to lose indication for surgery. 3) Those with unknown data about follow-up time, survival information or other characteristics. The enrolled subjects were allocated into a training cohort to develop a nomogram and an internal validation cohort randomly by 2 to 1 ratio.

### Study variables

The following variables of each patient were gathered: age, gender, carcinoma location, carcinoma grade, carcinoma size, AJCC TNM stage, regional lymph node examined, regional lymph node positive, lymph nodes surgery scope, and survival information. Regional lymph node positive was divided by regional lymph node examined to calculate the LNR value. The primary endpoint was OS with the definition of the duration from the diagnosing date until death due to any cause or last follow-up. The stage of carcinoma was identified by the TNM staging system of AJCC (7th edition). Patients in this study were limited to between 2010 and 2015 in consideration of this staging system having been accessible since 2010.

### Statistical analysis

All statistical tests were conducted using R project v. 3.5.2(The R Foundation for Statistical Computing, Vienna, Austria. http://www.r-project.org) and SAS v. 9.2 (SAS Institute Inc., USA). Categorical data were presented as frequency and percentage and tested with Chi-square test. Continuous data were expressed as the median and range and compared by Mann-Whitney U test. The optimal value of cutoff for LNR was decided using the analysis of time-dependent receiver operating characteristic (ROC) curve. Cox proportional hazards regression model was adopted to conduct the univariate and multivariate analysis, and we calculated the hazard ratio (HR) together with corresponding 95% confidence interval (CI). The OS were estimated by the Kaplan-Meier method and the test of log-rank was applied to analyze different survival curves. All analysis in this study was performed two-sided at the 5% significance level.

The rms package within R was applied to construct a nomogram on the basis of independent determinants identified in the multivariate Cox regression. The nomogram performance was judged using concordance index (C-index) and assessed by calibration curves as previously described [18]. The C-index value fluctuated from 0.5 to 1.0 with 0.5 representing random opportunity and 1.0 denoting a completely exact discrimination. The calibration curves from study cohort (bootstrap with 300 resamples) were applied to compare the concordance between the observed OS and the predicted OS probability.

## Results

### Characteristics of patients

In total, 6341 eligible patients over 40 years old with surgically resected PC from 2010 to 2015 were finally enrolled as the primary cohort, in which a training cohort and an internal validation cohort had 4242 patients and 2099 ones, respectively. The demographic and clinicopathological characteristics of patients were listed in Table 1. There was no statistically significant difference with respect to all the demographic and clinicopathological characteristics between training set and validation set. The median diagnosing age was 65 years old (range: 40–85 years old) in the whole patient cohort and age difference was not observed between training set and validation set. Totally, 3260(51.4%) were male, and the most common carcinoma location was pancreatic head (4750, 74.9%). The most common carcinoma grade was moderately differentiated (2866, 45.2%), then was poorly differentiated (2028, 32.0%). The majority of patients (5268, 83.1%) were classified as TNM stage II, followed by stage I (1073, 16.9%). Patients with 4 or more regional lymph nodes removed accounted for 5925 (93.4%). The primary cohort comprised 3195(50.4%)patients with carcinoma size of 2–4 cm, 1914 (30.2%) patients and 1232 (19.4%) patients with ≥4 cm and ≤ 2 cm, respectively. LNR was associated to the optimal Youdex index for predicting OS with 0.1732 being the cutoff value. The low-risk cohort (LNR ≤0.1732) consisted of 4348 (68.6%) patients.

**Table 1:** Demographical and clinicopathological characteristics of patients over 40 years old with surgically resected PC

Variable	Variable level	*N*	Training Set (*n* = 4242) *n*(%)	Validation Set (*n* = 2099) *n* (%)	*p-*value
Age (years)	< 60	1849	1231 (29.0)	618 (29.4)	0.727
	≥60	4492	3011 (71.0)	1481 (70.6)	
Gender	Male	3260	2196 (51.8)	1064 (50.7)	0.419
	Female	3081	2046 (48.2)	1035 (49.3)	
Tumor Location in pancreas	Head	4750	3174 (74.8)	1576 (75.1)	0.778
	Body	605	400 (9.4)	205 (9.8)	
	Tail	986	668 (15.8)	318 (15.1)	
Grade	Well differentiated	1350	928 (21.9)	422 (20.1)	0.433
	Moderately differentiated	2866	1898 (44.7)	968 (46.1)	
	Poorly differentiated	2028	1352 (31.9)	676 (32.2)	
	Undifferentiated	97	64 (1.5)	33 (1.6)	
AJCC TNM stage	I	1073	713 (16.8)	360 (17.2)	0.732
	II	5268	3529 (83.2)	1739 (82.8)	
Regional lymph nodes surgery	None	42	31 (0.7)	11 (0.5)	0.332
	1~3	374	240 (5.7)	134 (6.4)	
	≥4	5925	3971 (93.6)	1954 (93.1)	
Tumor size (cm)	≤2	1232	854 (20.1)	378 (18.0)	0.128
	(2~4)	3195	2123 (50.0)	1072 (51.1)	
	≥4	1914	1265 (29.9)	649 (30.9)	
LNR	≤0.1732	4348	2926 (69.0)	1422 (67.7)	0.321
	> 0.1732	1993	1316 (31.0)	677 (32.3)	

*Abbreviations: PC* pancreatic carcinoma, *AJCC* American Joint Committee on Cancer, *TNM* Tumor-Node-Metastasis, *LNR* lymph node ratio

### Univariate and multivariate analysis of determinants of OS

In total, the median follow-up time and median OS was 31 months (range: 1–71) and 25 months (95% CI: 23.95–26.05), respectively. The one-, two-, three- year rates of OS were 73.7, 50.8 and 37.7%, respectively. Totally, 3103/6341(48.9%) patients died, in which 2742 cancer-specific deaths and 361 non-cancer-specific deaths were observed, respectively. With regard to non-cancer-specific death, the top three most common causes were heart disease (69, 19.1%), septicemia (24, 6.7%) and cerebrovascular disease (18, 5.0%). As univariate test for training cohort showed, age, carcinoma location in pancreas, carcinoma grade, TNM stage, carcinoma size and LNR observed statistically significant associations with OS (*P* < 0.01), while gender and regional lymph nodes surgery did not meet the prespecified threshold for statistical significance with OS (*P* > 0.05) (Table 2).

**Table 2:** Univariate and multivariate analysis of factors associated with OS of patients in the training cohort

Variable	Variable level	Univariate analysis			Multivariate analysis		
		HR	95%CI	*p-*value	HR	95%CI	*p-*value
Age	< 60	Reference			Reference		
	≥60	1.517	1.370~1.680	< 0.001	1.328	1.198~1.471	< 0.001
Gender	Male	Reference					NI
	Female	0.968	0.888~1.056	0.470			
Tumor Location in pancreas	Head	Reference			Reference		
	Body	0.607	0.513~0.719	< 0.001	0.875	0.737~1.038	0.124
	Tail	0.507	0.438~0.588	< 0.001	0.737	0.634~0.857	< 0.001
Grade	Well	Reference			Reference		
	Moderately	3.495	2.983~4.094	< 0.001	2.616	2.224~3.078	< 0.001
	Poorly	5.210	4.437~6.118	< 0.001	3.584	3.034~4.233	< 0.001
	Undifferentiated	4.274	3.001~6.086	< 0.001	3.385	2.371~4.832	< 0.001
AJCC TNM stage	I	Reference			Reference		
	II	3.661	3.081~4.351	< 0.001	1.855	1.542~2.231	< 0.001
Regional lymph nodes surgery	None	Reference					NI
	1~3	1.477	0.600~3.638	0.396			
	≥4	2.198	0.914~5.286	0.079			
Tumor size (cm)	≤2	Reference			Reference		
	(2~4)	1.960	1.715~2.240	< 0.001	1.303	1.136~1.494	< 0.001
	≥4	2.200	1.911~2.533	< 0.001	1.512	1.307~1.749	< 0.001
LNR	≤0.1732	Reference			Reference		
	> 0.1732	1.991	1.821~2.176	< 0.001	1.522	1.388~1.669	< 0.001

*Abbreviations*: *OS* overall survival, *AJCC* American Joint Committee on Cancer, *TNM* Tumor-Node-Metastasis, *LNR* lymph node

**Fig. 1:**
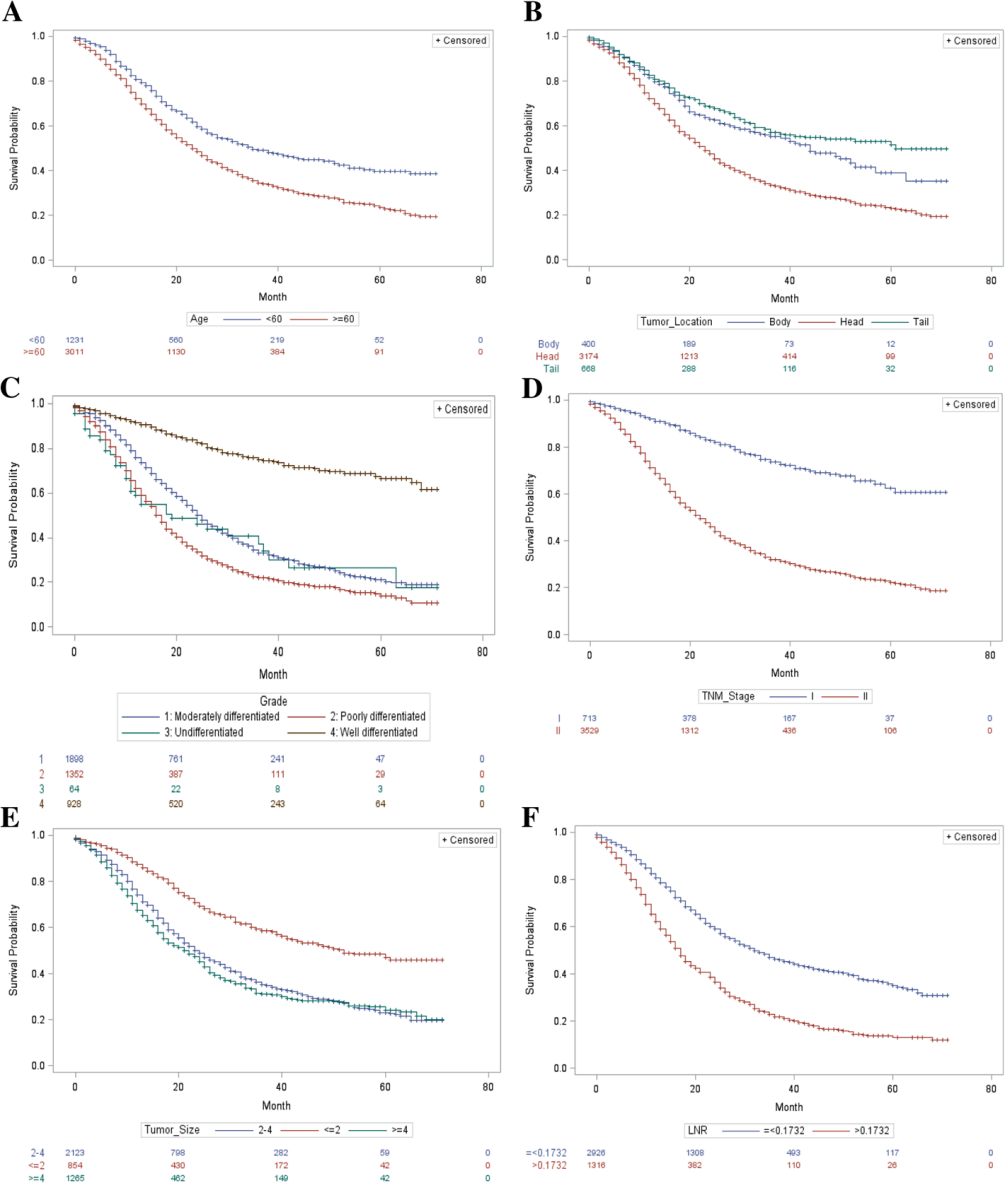
Kaplan-Meier OS curves stratified by patient characteristics: (**a**)Age; (**b**) Pancreatic Location; (**c**) Tumor Grade; (**d**) TNM 7th stage; (**e**) Tumor Size; (F)LNR. *Abbreviations*: *TNM* Tumor-Node-Metastasis, *LNR* lymph node ratio

For multivariate Cox regression model, a backward stepwise procedure was performed after selecting all the variables identified by the univariate model as potentially prognostic determinants. Additionally, in view of TNM stage probably being relevant to tumor size and the presence of lymph node metastasis, the possible interaction between TNM stage and tumor size, together with interaction between TNM stage and LNR, were also incorporated into the multivariate model. Multivariate analysis demonstrated that 6 determinants involving age, carcinoma location in pancreas, carcinoma grade, stage of TNM, carcinoma size and LNR remained as independent survival predictors associated with OS (Table 2). None of interactions were found to be statistically significant in their effects on overall survival. Patients with elder age (HR = 1.328, 95% CI: 1.198–1.471), advanced grade (HR = 2.616 for moderately differentiated, 95% CI: 2.224–3.078; HR = 3.584 for poorly differentiated, 95% CI: 3.034–4.233; HR = 3.385 for undifferentiated, 95% CI: 2.371–4.832), advanced stage of TNM (HR = 1.855 for II stage, 95% CI: 1.542–2.231), enlarged carcinoma (HR = 1.303 for 2-4 cm, 95% CI: 1.136–1.494; HR = 1.512 for ≥4 cm, 95% CI: 1.307~1.749) and LNR larger than 0.1732 (HR = 1.522, 95% CI: 1.388–1.669) suffered from more inferior survival. While patients with carcinoma location in the pancreatic body (HR = 0.875, 95% CI: 0.737–1.038) and carcinoma location in the pancreatic tail (HR = 0.737, 95% CI: 0.634–0.857) were more likely to experience better survival compared with those whose primary tumors were located in pancreatic head. Beyond that, survival curves of Kaplan-Meier demonstrated the OS differences with respect to stratification by these factors were all statistically significant (Fig. 1).

### Constructing and validating nomogram for OS

**Fig. 2:**
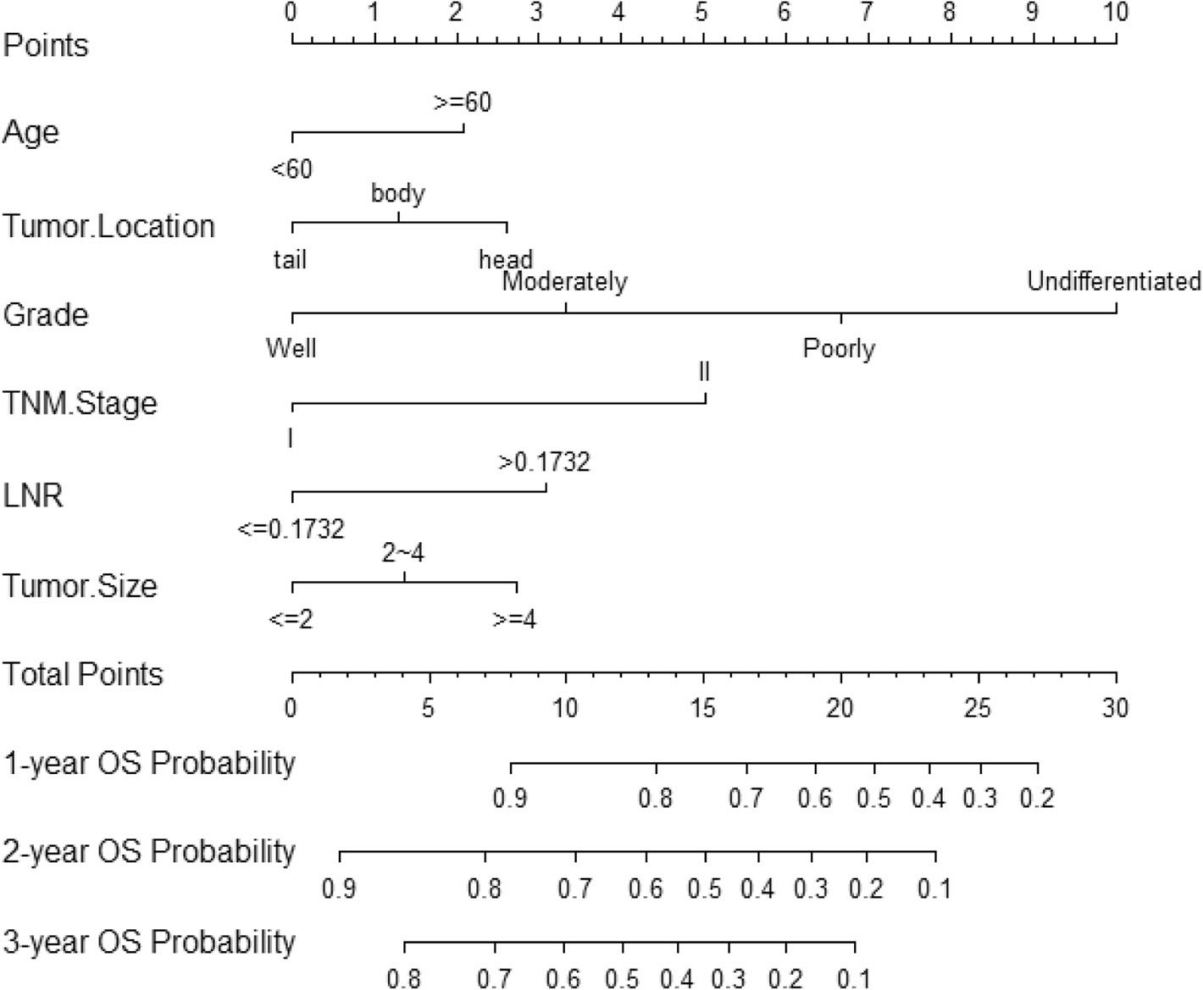
Nomogram for predicting 1-, 2-, 3-years OS of patients The nomogram is used by adding the points identified on the scale for 6 variables to achieve the total points, and a vertical line is drawn downward to the survival axes to determine the probability of 1-,2- and 3-years OS. Abbreviations: *TNM* Tumor-Node-Metastasis, *LNR* lymph node ratio, *OS* overall survival.

**Fig. 3:**
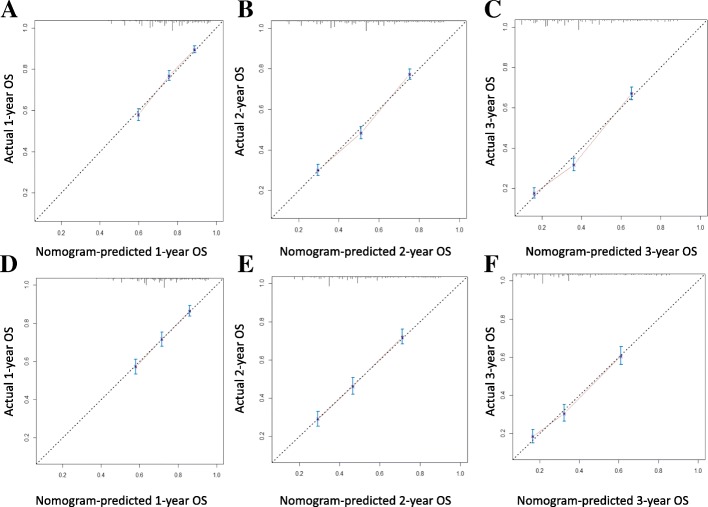
Calibration plots of nomogram for 1-, 2- and 3-year OS prediction of the training set (**a**, **b**, **c**) and validation set (**d**, **e**, **f**) X-axis represents the nomogram-predicted OS probability and Y-axis represents the actually observed OS probability. The diagonal line indicates the perfect nomogram reference. Dots with bars represent nomogram-predicted probabilities together with 95% confidence interval.

All of prognostic determinants identified from training set were brought into the construction of the nomogram. Figure 2 could illustrate a nomogram from training cohort which was constructed for the one-, two-, and three- year probabilities of OS. An individual patient’s survival probability may be simply obtained by summing the point of each factor on the points scale to get the total point score, then, the total score is matched vertically downward to the scale of survival to determine the probability. Took 2 stage II PC patients for example (Table 3): the first case with 55-years old was diagnosed with a poorly differentiated tumor of 4 cm in pancreatic head, and the second case who was 65-years old was diagnosed with a moderately differentiated tumor of 4 cm in pancreatic tail. Meanwhile, both of them suffered from a LNR > 0.1732. Using nomogram, those 2 cases had the total points of 20 and 16 respectively, and achieved one-year OS probability of 55 and 72%, respectively. The nomogram showed a well discriminatory precision with the C-index being 0.680(95%CI: 0.667–0.693). Calibration curves showed an excellent unanimity between the actually observed and nomogram-predicted survival for one-, two-, and three- year OS in two sets (Fig. 3).

**Table 3:** Comparison of two AJCC TNM stage II PC patients according to variables in Nomogram and 1-year OS

Variable	Patient 1			Patient 2		
	Value	Points	1-year OS	Value	Points	1-year OS
Age	55	0		65	2	
Tumor Location in pancreas	Head	2.5		Tail	0	
Grade	Poorly	6.75		Moderately	3.25	
AJCC TNM stage	II	5		II	5	
Tumor size (cm)	4	2.75		4	2.75	
LNR	> 0.1732	3		> 0.1732	3	
Total Points		20	55%		16	72%

*Abbreviations*: *OS* overall survival, *AJCC* American Joint Committee on Cancer, *TNM* Tumor-Node-Metastasis, *LNR* lymph node

### Survival analysis by risk stratification on the basis of nomogram

**Fig. 4:**
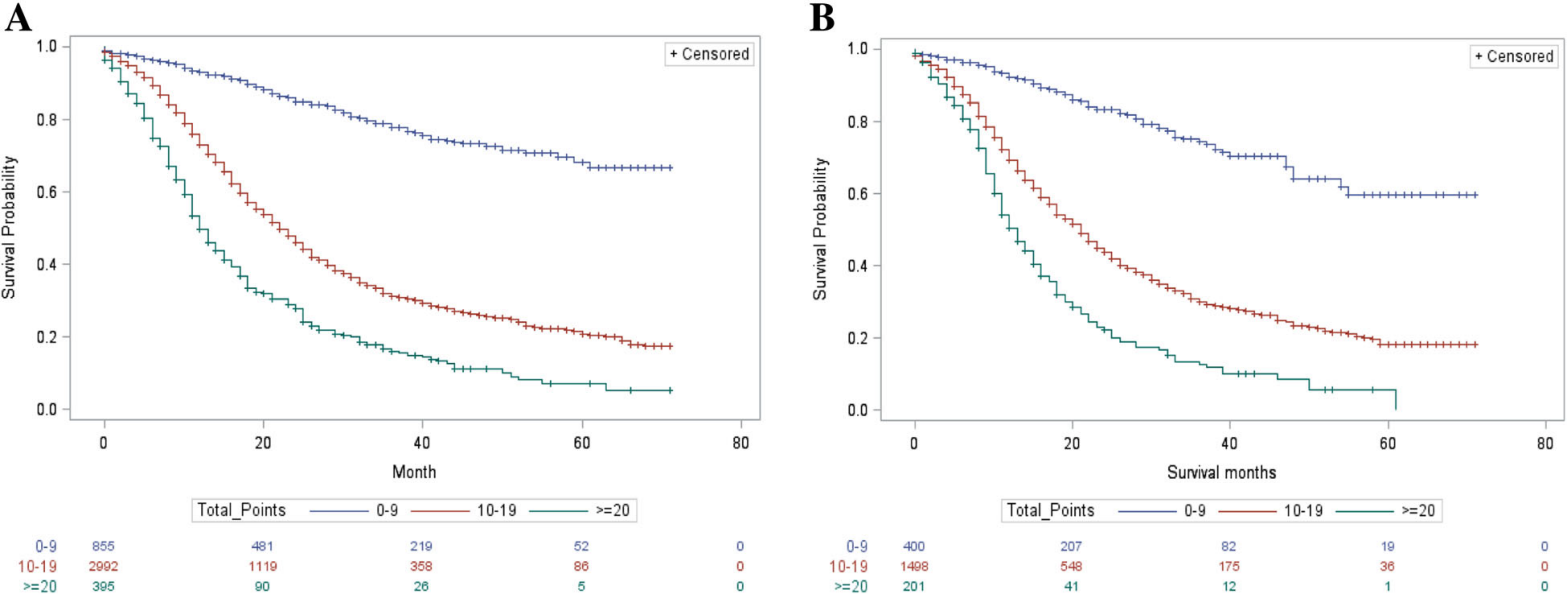
Kaplan-Meier OS curves according to the risk levels of nomogram-predicted survival probabilities: (**a**) Training set; (**b**) Validation set

Patients in two sets were categorized into low, middle and high risk cohorts by the total points derived from the nomogram. Those subjects with total points of greater than or equal to 20, 10–19, and less than 10 were identified as the high, middle, and low risk group, respectively. The survival curves of Kaplan-Meier according to risk stratification were demonstrated in Fig. 4. Compared with patients in the high risk group, patients in the rest of two risk groups showed more significantly superior OS rates not only in training set but also in validation set.

## Discussion

Several previously reported nomograms for PC patients were based on either limited variables and comparatively small sample size, or no limitation of age, or being irrespective of surgery status [19–21]. Therefore, developing and validating a nomogram for PC with better applicability is still needful. In this study, 6341 patients greater than 40 years old with surgically resected PC were enrolled from the SEER dataset and analyzed to build the OS-predicting nomogram. Six independent prognostic determinants invloving age, carcinoma location in pancreas, size of carcinoma, grade, stage of TNM together with LNR were identified through the univariate and multivariate Cox proportional hazard regression. A nomogram based on these factors was constructed and manifested favorable discrimination and calibration, which meant it might act as a quantitative model to appraise individual OS rate of PC patients.

It appears that age has been a vital prognostic determinant. Within our study which included the patients older than 40 years old, multivariate analysis signified that elder age had a straightforward impact on OS. Further stratified survival analysis manifested patients older than 60 years old had a more inferior survival in comparison with patients with age fallen between 40 and 60 years old. This result resembled other studies which reflected that increasing age might contribute to mortality of patients [20, 21]. Approximately 80 % of all PC occur in pancreatic head, and prognosis of this type of carcinoma continues to be inferior even experiencing pancreaticoduodenectomy which has ten to twenty months of median OS [22]. According to our analysis of Cox regression and log-rank test, patients with carcinoma location in pancreatic head were more likely to experience poorer survival, which was in accordance with the conclusion of Song’s study [20]. Grade of carcinoma demonstrates the biological behavior of neoplasm, which is highlighted for its significant impact on prognosis. It has been indicated that carcinoma differentiation is an independent determinant for predicting OS in similar researches [10, 22], and our multivariate analysis also showed poorer survival when carcinoma grade shifted to poor differentiation from well differentiation. Based on present nomogram, patients who had different carcinoma grades were given disparate scores and could get diverse survival probability, even though they were sorted into the same stage of TNM. This result clearly exhibited the difference between prognosis derived from traditional TNM staging system and those by nomogram. Considering the above-mentioned example, the two stage II PC patients with different age, pancreatic tumor location and grade suffered from different 1-year OS probability using nomogram. However, according to TNM staging system, both of them were identified as stage II, which indicated the same consequence. Superiority of nomogram in predicting survival compared with TNM staging system could be explained in part. As indicated in this study, TNM stage and tumor size were also involved in the formulation of nomogram, which were in accordance with past studies that signified the independent impact of the two indicators on OS predicting [20, 23]. Patients with advanced TNM stage and enlarged tumor suffered from higher mortality and poorer survival rate as demonstrated by multivariate Cox regression analysis. As far as we know, LNR value integrates information with respect to positive lymph nodes and total examined lymph nodes. Several studies had shown that increased LNR indicated the potential trend of progression or metastasis and revealed notoriously poorer prognosis [13, 24, 25]. By treating LNR, a continuous variable, as binary categorical variable with the cutoff value of 0.1732 at present study, patients could be easily divided into groups with different risks. Our nomogram allowed a simple and visually friendly means for survival prediction. We found that high LNR value exhibited to be a poorer prognostic indicator for OS, which was similar to the result of significant relationship between low distant metastasis-free survival and elevated LNR level (greater than 0.15) derived from MD Anderson Cancer Center [26]. As with previous study [21], we did not identify the amount of regional lymph nodes surgery as an OS determinant in patients with PC. It can be conjectured LNR value is an excellent indicator for prediction of survival outcome in comparison to the number of regional lymph nodes surgery. AJCC recommends 12 harvested lymph nodes, at a minimum, is sufficient for precisely classifying carcinoma staging as inadequate lymph nodes may result in understaging the N category in PC [27].

At present study, all indicators embodied in the nomogram were significant determinants of OS prediction among patients over 40 years old with surgically resected PC. Our nomogram showed good discrimination with C-index being 0.680. The calibration curve of both training set and internal validation set indicated goodness of fit in predicting survival since the OS at one-, two-, three-years predicted by nomogram were highly proximate with actual ones, respectively. Furthermore, survival curves stratified by nomogram-predicted survival risk probabilities demonstrated the statistically significant difference both in training cohort and validation one. Our nomogram which was constructed based on the large population of SEER database could embody more generalized applicability. Meanwhile, the nomogram incorporating variables that govern carcinoma prognosis can emerge as a simpler, more sophisticated tool to estimate individual survival risk, and may assist physicians in more accurate prognostic predicting and decision making concerning individual treatment.

Several limitations existed in our study. Firstly, patients were randomly allocated into training cohort for developing nomogram and internal validation cohort for assessing accuracy of nomogram. Though nomogram of present study exhibited perfect performance in OS predicting, validation using other external data is still required to undergo rigorous scrutiny and further evaluate predictive accuracy. Next, some other variables related to prognosis such as CA19–9 [28], the most extensively adopted serum indicator in PC prognosis, and vascular invasion [29] were unaccessible from SEER database. Study covering these variables will be the future research direction. Moreover, this study was based on retrospective data, the large-scaled and prospective study is still needed to eliminate the bias and validate the accuracy of nomogram. Only in this way can nomogram enable perfect prognostication for patients.

## Conclusions

We analyzed the clinicopathological factors determining OS of patients over 40 years old with surgically resected PC using a population-based SEER database. Furthermore, nomogram for predicting one-, two-, and three- years OS was developed. Our nomogram demonstrated good performance and can be considered as a novel assessing tool of individual survival.

## Data Availability

We obtained consent to acquire the database with SEER ID: 10930-Nov2017. The datasets used and/or analyzed during this study are available from the corresponding author on reasonable request.

## Abbreviations

AJCC: American Joint Commission on Cancer
CA19–9: Carbohydrate antigen 19–9
CI: Confidence interval
HR: Hazard ratio
ICD-O-3: International Classification of Diseases for Oncology, 3rd Edition
LNR: Lymph node ratio
NCI: National Cancer Institute
OS: Overall survival
PC: Pancreatic carcinoma
ROC: Receiver operating characteristic
SEER: Surveillance, Epidemiology, and End Results
TNM: Tumor-Node-Metastasis
